# Thermal Stability Threshold for Amyloid Formation in Light Chain Amyloidosis

**DOI:** 10.3390/ijms141122604

**Published:** 2013-11-15

**Authors:** Tanya L. Poshusta, Nagaaki Katoh, Morie A. Gertz, Angela Dispenzieri, Marina Ramirez-Alvarado

**Affiliations:** 1 Department of Biochemistry and Molecular Biology, Mayo Clinic, 200 First St. SW, Rochester, MN 55905, USA; E-Mail: Poshusta.tanya@mayo.edu; 2 Department of Medicine (Neurology and Rheumatology), Shinshu University, 3-1-1, Asahi, Matsumoto City 390-8621, Japan; E-Mail: nagaaki@shinshu-u.ac.jp; 3 Division of Hematology, Mayo Clinic, 200 First St. SW, Rochester, MN 55905, USA; E-Mails: gertz.morie@mayo.edu (M.A.G.); dispenzieri.angela@mayo.edu (A.D.)

**Keywords:** light chain amyloidosis, immunoglobulin light chain, hematologic response, organ response, thermodynamic stability, amyloid fibril formation, partially folded states

## Abstract

Light chain (AL) amyloidosis is a devastating disease characterized by amyloid deposits formed by immunoglobulin light chains. Current available treatments involve conventional chemotherapy and autologous stem cell transplant. We have recently concluded a phase III trial comparing these two treatments. AL amyloidosis patients who achieve hematological complete response (CR) do not necessarily achieve organ response regardless of the treatment they received. In order to investigate the possible correlation between amyloid formation kinetics and organ response, we selected AL amyloidosis patients from the trial with kidney involvement and CR after treatment. Six patients were selected and their monoclonal immunoglobulin light chains were characterized. The proteins showed differences in their stability and their kinetics of amyloid formation. A correlation was detected at pH 7.4, showing that less stable proteins are more likely to form amyloid fibrils. AL-T03 is too unstable to form amyloid fibrils at pH 7.4. This protein was found in the only patient in the study that had organ response, suggesting that partially folded species are required for amyloid formation to occur in AL amyloidosis.

## 1. Introduction

Light chain (AL) amyloidosis is a rare, devastating protein misfolding disease characterized by the abnormal proliferation of plasma cells that secrete monoclonal immunoglobulin light chains (either κ or λ) that misfold and aggregate as amyloid fibrils in vital organs, causing tissue degeneration and organ failure [1]. AL Amyloidosis is difficult to diagnose because there is no single test that is diagnostic for this disorder. In addition, the presenting symptoms can be very broad and are often mimicked by more common disorders. The diagnosis of AL amyloidosis should be suspected in patients with non-diabetic nephrotic syndrome; nonischemic cardiomyopathy with hypertrophy on echocardiography; hepatomegaly or increased alkaline phosphatase value with no imaging abnormalities of the liver; chronic inflammatory demyelinating polyneuropathy with a monoclonal light chain protein; or presence of a monoclonal gammopathy in a patient with unexplained fatigue, edema, weight loss, or paresthesias (reviewed in [2,3]). The median survival for AL amyloidosis patients is 12–40 months [4]. The most frequently affected organs are the kidneys, the heart, liver, and peripheral nerves. Current treatments target the malignant plasma cell clone and include standard chemotherapy and autologous stem cell transplant (SCT). AL amyloidosis is not only a hematological malignancy as patients also have multiorgan dysfunction, which determines prognosis and renders them more susceptible to treatment toxicity [5]. SCT-related mortality rates have decreased from as high as 40% to 7% in current studies. The major limitation to wider application of SCT is that no more than 20%–25% of patients are eligible for transplant. Currently, a hematologic response is achievable in 76% of eligible patients, and a complete response in 39% [3]. The goal of treatment is a reduction in the concentration of the circulating free light chain (FLC), defined as hematological response. AL patients who achieved hematological complete response (CR) (after day 100, post transplant) do not necessarily achieve organ response regardless of the treatment they received. Organ response is defined based on the organ involvement and is evaluated every six months for the first three years post treatment.

The criteria utilized for the Phase III clinical trial at Mayo Clinic comparing standard chemotherapy *vs.* autologous stem cell transplant are the following: Renal response was defined by 50% or more reduction in 24 h urine protein excretion (at least 0.5 g/day). Creatinine and creatinine clearance must not worsen by 25% over baseline (minimum change of creatinine 0.5 mg/dL and of creatinine clearance 15 mL/min).

In this study, we have compared proteins from AL amyloidosis patients who have achieved hematologic response and present predominant renal involvement. We have compared the stability and kinetics of amyloid formation of their corresponding immunoglobulin light chain variable domains. Our hypothesis was that there will be a correlation between thermal stability and kinetics of amyloid formation among AL proteins. We found that there is indeed a correlation between thermal stability and amyloid formation. Surprisingly, we found that the only patient with organ response produces the most thermally unstable protein and which is unable to form amyloid fibrils under physiologic solution conditions. Our results suggest that there is a thermal stability threshold required for amyloid formation in AL amyloidosis.

## 2. Results and Discussion

**Figure 1:**
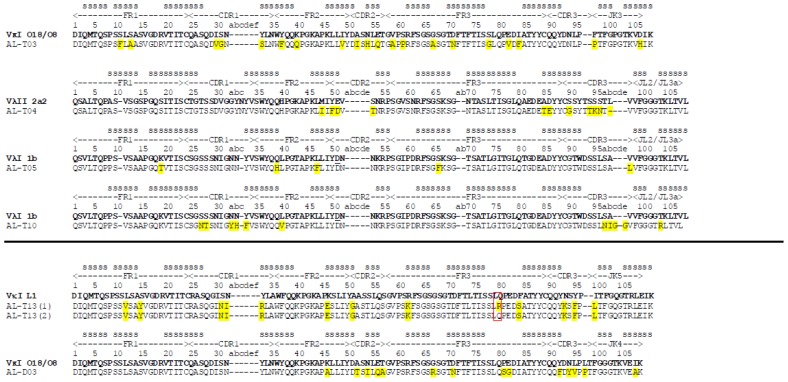
Protein sequence alignment corresponding to the variable domain of immunoglobulin light chains from light chain (AL) amyloidosis patients characterized in this study. The somatic mutations are shown as yellow highlights. The red box indicates the amino acid difference between AL-T13 (1) and AL-T13 (2). Each protein has been aligned with its corresponding germline donor sequence (using the Kabat nomenclature) and the secondary structure (s = β-strand) is shown from known crystallographic data as previously reported [7].

Six patients fulfilled the criteria for inclusion in this study (renal involvement, hematologic CR). We isolated RNA from their bone marrow plasma cells, and their pathogenic immunoglobulin light chain variable regions were sequenced (Table 1). The patients’ samples were labeled with AL (light chain amyloidosis) T or D. T for transplant arm of the trial; D for drug arm of the trial (chemotherapy) and a number (01–99). Three patients (AL-T03, ALT-13 and AL-D03) presented a dominant clone from a κ germline sequence (two were VκI O18/O8 (*Kabat nomenclature*) (IGKV 1–33, *IMGT nomenclature*) and one corresponded to VκI L1 (IGKV 1–16)). The remaining three patients (AL-T04, ALT-05, ALT-10) had a dominant clone from a lambda germline sequence (one from Vλ2 2a2 (IGLV 2–14); two from Vλ1 1b (IGLV 1–51)). We were unable to determine a single identical (monoclonal) light chain sequence for one patient (AL-T13). This patient presented an almost identical cDNA sequence except for one codon corresponding to the amino acid residue 79. About 50% of the sequences presented Arg in position 79, while 50% presented the amino acid Gln 79. We decided to limit our study to those patients with a monoclonal population, thus, this patient’s protein was not included in the subsequent analysis. Figure 1 shows the protein sequences derived from cDNA sequencing of plasma cells from the six patients included in this study, including the two versions of patient AL-T13 found in our analysis.

The five remaining proteins were expressed in recombinant bacterial cultures and purified from inclusion bodies. The protein AL-D03 was not successfully purified in the folded state, possibly due to a putative glycosylation site acquired with a somatic mutation (The somatic mutation Asn 70 generated the putative glycosylation site N-X-S/T [6]: N70-F71-T72). Bacterial expression does not allow post-translational modifications to occur, and it is not clear if the possible glycosylation that could be occurring in this protein is necessary for the appropriate folding of this protein. Therefore, we did not continue our analysis with AL-D03. The remaining four proteins were purified and characterized for their global overall structure using circular dichroism spectroscopy.

### 2.1. AL Proteins from Different Patients Adopt an Overall β-Sheet Structure

**Figure 2:**
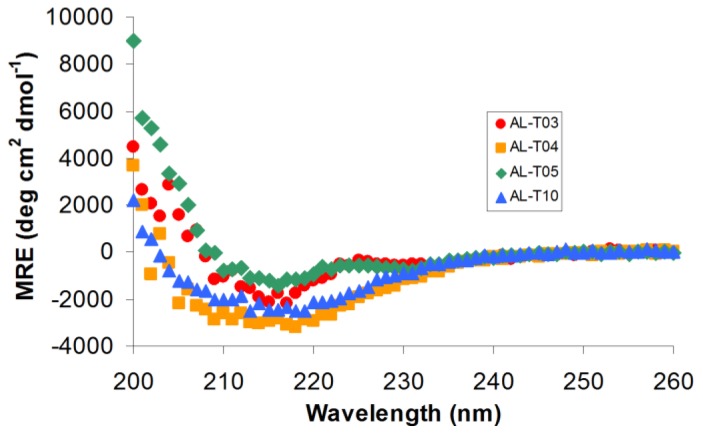
Far UV CD spectra of AL proteins characterized in this study. The experiments were performed in 10 mM Tris–HCl pH 7.4 at 4 °C. The minimum mean residue ellipticity (MRE) at 217 nm indicates that the proteins adopt the expected β-sheet structure typical of immunoglobulin domains.

We analyzed the remaining four AL proteins using Far UV-Circular Dichroism (CD) spectroscopy to determine the overall structure adopted by these proteins. AL-T03 and AL-T05 presented two minima in their Far UV-CD spectra: one around 235 nm (indicative of aromatic residues that are optically active in the Far UV region) and one around 217 nm (indicative of β-sheet structure), typical of light chain variable domain proteins, as has been extensively reported previously by our laboratory and others [8–12]. AL-T04 and AL-T10 CD spectra present only one broad minimum around 217 nm. The mean residue ellipticity value is very similar for all of the proteins around 217 nm as shown in Figure 2.

### 2.2. AL Proteins from Different Patients Present Different Thermal Stability

**Figure 3:**
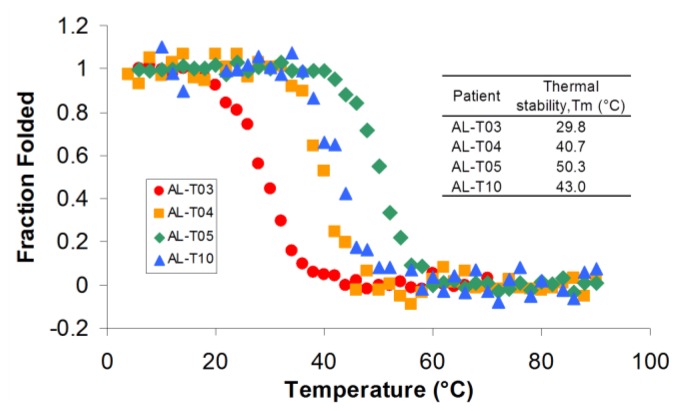
AL proteins present significant differences in thermal stability. The experiments were performed in 10 mM Tris–HCl, pH 7.4. The ellipticity at 217 nm was followed as a function of temperature with a scan rate of 30 °C per hour.

Once we determined that the proteins were properly folded, we characterized their thermal stability by following the ellipticity at 217 nm as a function of temperature (Figure 3). All proteins showed the typical two-state unfolding transition and were able to refold reversibly. AL-T10 showed a slight hysteresis in the refolding (cooling) curve as it refolded reversibly, possibly due to a kinetic control refolding process as we have recently described [13]. The remaining four proteins characterized in this study presented significant differences in thermal stability. AL-T04 and AL-T10 exhibit a melting temperature (*T*m or the temperature in which 50% of the protein is folded) that is similar to the value reported for other AL proteins characterized in our laboratory. AL-T04 belongs to the germline Vλ2 2a2 (IGLV 2–14) and AL-T10 belongs to Vλ1 1b (IGLV 1–51), while the proteins that our laboratory has characterized so far belong to Vκ1 O18/O8 (IGKV 1–33) (reviewed in [14]). This suggests that the germline donor sequence does not determine the thermal stability of the protein but rather the somatic mutations in specific locations in the protein structure cause the loss of stability in these amyloidogenic proteins [7]. AL-T03 is the most unstable protein of this group with a *T*m of 29.8 °C, and to the best of our knowledge, the most unstable amyloidogenic light chain protein ever characterized to date. AL-T03 belongs to Vκ1 O18/O8 (IGKV 1–33) and presents 20 somatic mutations, 16 of which are non-conservative. In contrast, AL-T05 is one of the most stable amyloidogenic light chain proteins characterized with a Tm of 50.3 °C. AL-T05 belongs to Vλ1 1b (IGLV 1–51) and presents five somatic mutations, four of which are non-conservative. AL-T05 and AL-T10 belong to the same germline donor sequence; AL-T10 has 10 somatic mutations, five of which are non-conservative, confirming the notion that the somatic mutations play a major role determining the thermodynamic stability of amyloidogenic light chain proteins.

### 2.3. Amyloid Fibril Formation Kinetics Correlates with Thermal Stability at pH 7.4

**Figure 4:**
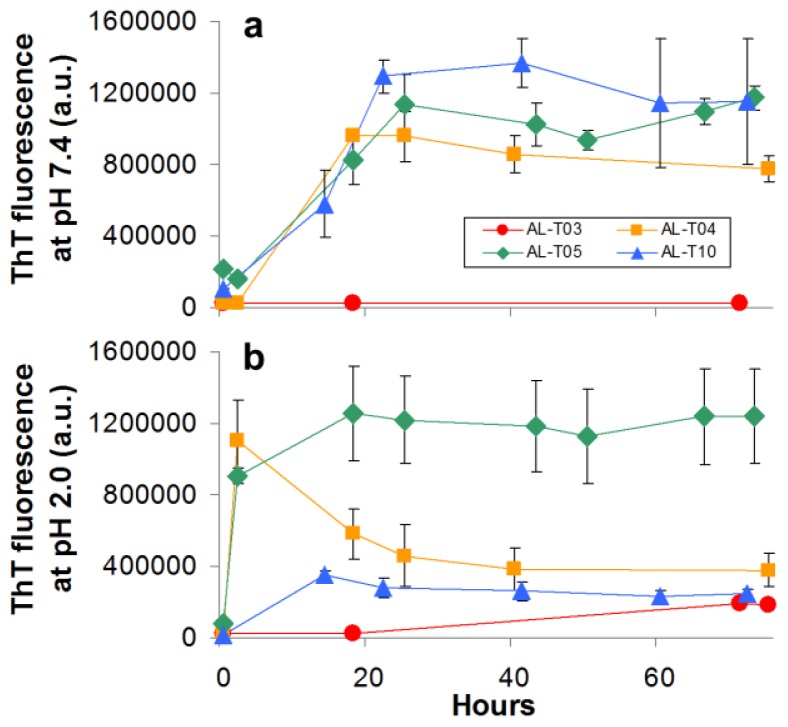
AL proteins show differences in their ability to form amyloid fibrils *in vitro*. Increase in ThT fluorescence over time indicates amyloid fibril formation. At pH 2.0, the proteins form amyloid fibrils faster than at pH 7.4. AL-T03 was only capable of forming amyloid fibrils at pH 2.0.

We performed amyloid formation reactions *in vitro* to compare how the kinetics of amyloid formation compared among the different AL proteins in our study. We conducted these *in vitro* experiments at different pH values, and at 37 °C, following amyloid formation with Thioflavin T fluorescence as described previously [15]. All proteins were ultracentrifuged prior to the fibril formation assay in order to remove preformed aggregates (the ultracentrifugation protocol used in this study enriches for monomeric species). The kinetics of amyloid formation show an exponential phase followed by a stationary phase. There is a short lag time, so the overall kinetic profile follows the typical sigmoidal curve observed for amyloid formation kinetics *in vitro* [16]. The kinetics of amyloid formation for the AL proteins studied in this report are slower at pH 7.4 than at pH 2.0, in agreement with what has been published by our group and others [9,10,15,17] as seen in Figure 4. Most proteins follow very similar kinetics of amyloid formation at pH 7.4 except AL-T03, which does not form amyloid fibrils at pH 7.4. At pH 2.0, AL-T05 and AL-T04 form fibrils almost within our detection time, AL-T10 is slightly delayed compared to these other two proteins and AL-T03 presents a modest, non-cooperative increase in the Thioflavin T fluorescence reaching the maximum fluorescence attained by AL-T10 after 60 h. It is worth noting that AL-T04 shows a rapid increase followed by a decrease in the Thioflavin T emission fluorescence. We have observed this phenomenon previously; it is possibly due to inner filter effects [15].

**Figure 5:**
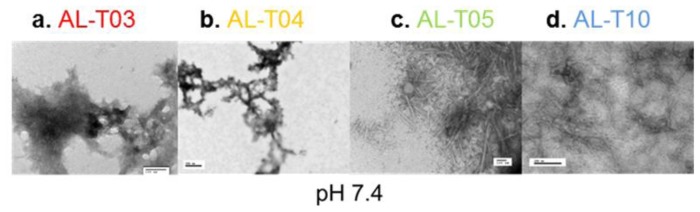

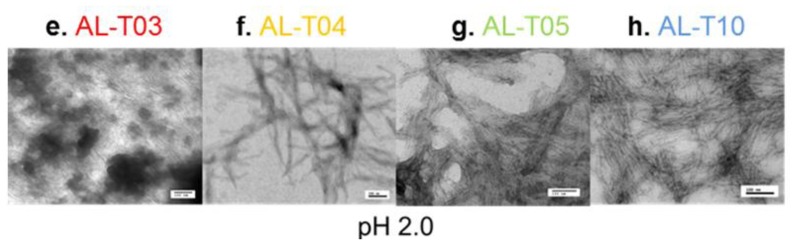
Transmission electron microscopy of representative amyloid fibrils (**a**–**d**) Amyloid formation reactions at pH 7.4; (**e**–**h**) Amyloid formation reactions at pH 2.0.

To confirm that the increase in Thioflavin T fluorescence is indeed associated with amyloid formation, we performed transmission electron microscopy on samples taken at the end of the kinetic reactions for all proteins at pH 7.4 and pH 2.0 (Figure 5). At pH 7.4, AL-T03 presents amorphous aggregates; AL-T04 presents a mixture of amorphous aggregates with amyloid fibrils, while AL-T05 and AL-T10 present typical amyloid fibrils formed by immunoglobulin light chains. At pH 2.0, AL-T03 shows the mixture of amorphous aggregates and amyloid fibrils in a slightly different morphology than the one observed for AL-T04 at pH 7.4, while AL-T04, AL-T05, and AL-T10 show typical amyloid fibrils. In the case of AL-T05 and AL-T10, the amyloid fibrils are abundant in the sample and present lateral fibril interactions.

**Figure 6:**
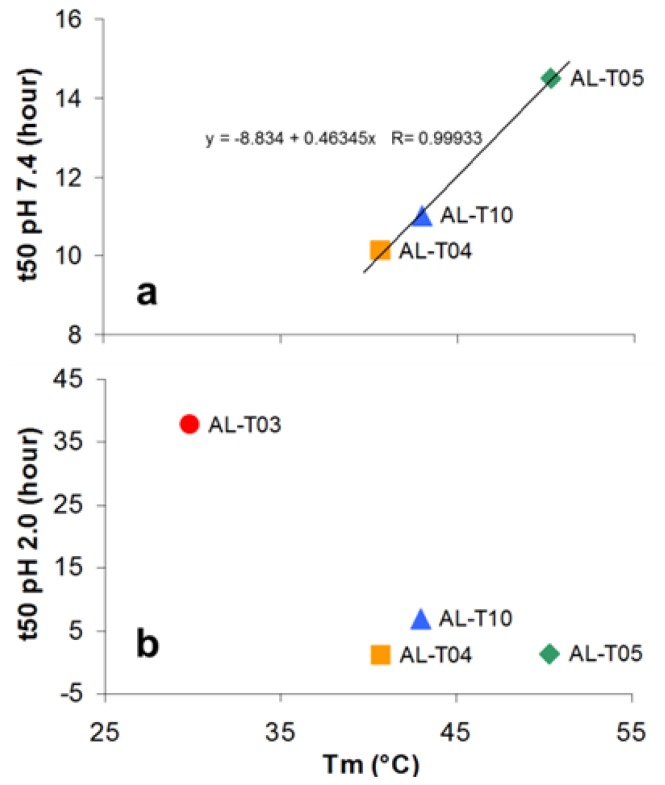
The relationship between *T*m and t50 (**a**) at pH 7.4 and (**b**) pH 2.0. Linear fitting was performed at pH 7.4 with the equation shown.

Based on the thermal stability and amyloid formation kinetics results, we wanted to determine if there is any correlation between thermodynamic stability and the propensity to form amyloid fibrils, as has been published by our laboratory and others (reviewed in [14]). Therefore, we plotted t50 values for fibril formation as a function of *T*m using the t50 values at pH 7.4 and at pH 2.0, respectively (Figure 6).

The three proteins that form fibrils at pH 7.4 present a clear correlation between their ability to form amyloid fibrils and their thermal stability. The more unstable proteins form fibrils faster than the stable proteins do. The situation is quite different at pH 2.0. The four proteins were able to form fibrils under this condition, but there is no clear correlation between the thermal stability and their ability to form amyloid fibrils.

Finally, we wanted to make another correlation between amyloid fibril formation kinetics *in vitro* and the organ response after treatment. All of these patients presented with hematologic complete response. The organ response data is the following:

AL-T03 ------ Renal response after one year post transplant
AL-T04 ------ No renal response after one year post-transplant
AL-T05 ------ No renal response after one year post-transplant
AL-T10 ------ No renal response after one year post-transplant

These results were quite surprising because they suggest that the most unstable protein in our study (AL-T03) came from the only patient in this group that presented renal response. There are numerous ways to interpret this result. One interpretation could be that AL-T03 has low thermodynamic stability (*T*m = 29.8 °C) below the required threshold for amyloid formation to occur under physiological conditions (37 °C, pH 7.4). AL-T03 is 70% unfolded at 37 °C, pH 7.4, conditions where it was unable to form amyloid fibrils *in vitro*, suggesting that partially folded species formed during the unfolding transition at pH 7.4 are required for amyloid formation to occur in AL amyloidosis, and AL-T03 is too unstable to populate these partially folded states at 37 °C.

The other interpretation could be that the rate of amyloid formation *in vitro* and organ response may be inversely correlated. If amyloid formation and the formation of toxic species come hand in hand, then the inability to form fibrils under physiological solution conditions may be associated with the inability to populate toxic species that cause disease progression and the absence of organ response after treatment. Our study has only been conducted with four proteins and the linear correlation only was performed with three points, so it is clear that in order to confirm the interpretations of this study, future studies are needed with other proteins presenting similar behavior.

Based on the electron micrographs and the fibril formation kinetic data, it would appear that the AL-T03 protein is more reminiscent of light chains found in patients with light chain deposition disease (LCDD). These patients present better organ response to therapy [18,19], in agreement with what we observe in this study. AL-T03 has two Proline somatic mutations (S60P and F96P) that will cause protein instability and would cause delays in amyloid fibril formation. It is possible that these two Proline residues play an important role in the behavior observed with this protein.

## 3. Experimental Section

### 3.1. Patient Eligibility for the Clinical Trial

i) Required Characteristics
  (a) Histologic proof of amyloidosis.
  (b) The amyloidosis must be of AL type. The patients must have a monoclonal protein by immunoelectrophoresis or immunofixation of the serum or urine or have an elevation in their free light chain ratio [20]. Secondary and familial amyloidosis must be excluded, and available tissues stained with appropriate anti-sera to confirm the type.
  (c) Typical amyloid syndromes considered eligible include amyloid hepatomegaly, cardiomyopathy, proteinuria, peripheral or autonomic neuropathy, or soft tissue involvement including the tongue, submandibular tissues, and vascular claudication. Occasional patients with diffuse interstitial pulmonary AL would be eligible if their pulmonary function is adequate to allow safe transplantation.
  (d) ≥18 years of age.
  (e) Eastern Cooperative Oncology Group (ECOG) performance status (PS) 0, 1, or 2.
  (f) The following laboratory values must be obtained ≤8 weeks of registration:
    - Platelets (PLT) ≥ 100,000/μL
    - Direct bilirubin ≤2.0 × ULN
    - Alkaline phosphatase ≤6 × ULN
    - Serum creatinine ≤3.0 mg/dL.
  (g) Must be previously untreated.
  (h) Must have a compensated cardiac status.
  (i) New York Heart Association classification I, II, or III.
ii) Contraindications
  (a) Multiple myeloma with lytic bone lesions or in excess of 30% plasma cells in the bone marrow.
  (b) Must not have amyloidosis that is manifest only by carpal tunnel syndrome or purpura. The presence of amyloid deposits in a plasmacytoma or in bone marrow vessels in an asymptomatic individual does not constitute an amyloid syndrome. Patients with overt multiple myeloma with lytic or destructive bone lesions or myeloma cast nephropathy.
  (c) Previous exposure to alkylating agents, immunosuppressive drugs, or steroids.
  (d) Infection at the time of registration.
  (e) HIV positive.
  (f) Any of the following:
    - Pregnant women
    - Nursing women
    - Men or women of childbearing potential who are unwilling to employ adequate contraception (condoms, diaphragm, birth control pills, injections, under-the-skin implants, intrauterine device [IUD], surgical sterilization, abstinence, *etc.*) This study involves an agent that has known genotoxic, mutagenic, and teratogenic effects.

### 3.2. Sample Collection

BM aspirates were collected from AL amyloidosis patients who enrolled in the Phase III clinical trial. Six patients were selected based on the following criteria: Dominant renal involvement and hematologic CR.

BM aspirates were treated with ammonium chloride buffer to lyse red cells and then CD138 positive cells were isolated using magnetic bead sorting. Total RNA was extracted and converted to cDNA.

### 3.3. Identification of the Clonal Ig Light Chain Variable Gene (V_L_)

The cDNA was amplified by PCR using 5′ primers specific for the FR1 region of Vλ or Vκ families and 3′ primers specific for the initial codons of the corresponding constant region. Bands were cut out, and the DNA was extracted and sequenced. Once its germline sequence was identified, the cDNA was amplified again by 2nd PCR using the leader specific 5′ primer and 3′ primers specific for the initial codons of the corresponding constant region, and the product was inserted into the pCRII TOPO vector plasmid. Cloned DNA was sequenced, and the most dominant clone in BM was determined. The cDNA corresponding to the variable domain and the junction region were then subcloned into pET12a vector for protein expression in *E. coli*. This vector contains the ompT leader peptide that directs the polypeptide towards the periplasmic space.

### 3.4. Protein Expression

Rosetta Gami *E. coli* cells were transformed with the corresponding plasmid and grown under the appropriate conditions for optimal protein expression for each protein (37 °C growth pre induction; 20–37 °C 4–20 h growth post induction). Small-scale extraction of periplasmic, cytoplasmic, and insoluble fractions determined the location of the protein in the bacterial cells. Cells were harvested, and the pellets were frozen at −20 °C. The thawed cell pellet was resuspended into 10 mM Tris buffer pH 7.4, and lysed by ultrasonication. Proteins were extracted from solubilized inclusion bodies using 6.0 M urea and immediately dialyzed overnight against 10 mM Tris–HCl (pH 7.4) to remove the urea. The overnight dialysis procedure provides the appropriate redox environment to form the disulfide bond between Cys 23 and Cys 88 in the extract [21]. AL-T05 was also found in the soluble fraction, so both soluble and insoluble fractions were purified. All protein extracts were filtered through 0.45 μm membranes and injected into a HiLoad 16/60 Superdex^®^ 75 size exclusion chromatography column on an AKTA FPLC (GE Healthcare, Piscataway, NJ, USA) system. AL-T10 was also purified using ion exchange chromatography, although the results were identical to those obtained with size exclusion chromatography except that ion exchange chromatography yielded less pure protein. Protein purity was determined by SDS-PAGE from the chromatographic peak corresponding to monomeric protein. Protein concentration was determined by UV absorption using an extinction coefficient calculated from the amino acid sequence. Pure fractions were combined, concentrated, flash frozen, and stored at −80 °C. Proteins were thawed at 4 °C, filtered and/or ultracentrifuged before they were used for each study.

### 3.5. Circular Dichroism Spectroscopy

Circular dichroism (CD) spectroscopy was used to determine the global secondary structure and the thermal stability after protein extraction/purification as described previously [9]. Briefly, far UV-CD spectra from 260–200 nm (1 nm bandwidth) were acquired at 4 °C in 10 mM Tris–HCl, pH 7.4, on a Jasco Spectropolarimeter 810 (JASCO, Inc., Easton, MD, USA) using a 0.2 cm path-length quartz cuvette. Thermal unfolding and refolding experiments were carried out following the ellipticity at 217 nm over a temperature range of 4–90 °C to determine the melting temperature (*T*m = the temperature where 50% of the protein is unfolded).

### 3.6. Amyloid Fibril Formation *In Vitro*

Fibril formation kinetics was investigated by measuring Thioflavin T (ThT) fluorescence. The proteins were ultracentrifuged for 25 h to remove preformed aggregates and to enrich for monomeric species to the sedimentation point of a 0.5S particle (90,000 rpm in a NVT-90 rotor on an Optima L-100 XP centrifuge (Beckman Coulter Pasadena, CA, USA) as reported previously [15]. The protein was placed on a polystyrene 96-well plate, incubated with ThT at 37 °C and shaken continuously at 300 rpm. For the reaction at pH 7.4, we used 10 mM Tris–HCl with 150 mM NaCl. For the reaction at pH 2, we used 10 mM sodium acetate, borate, and citrate. The ThT signals were monitored at different times, and the time to reach 50% maximum ThT fluorescence (t50; the time where fibril formation is 50% completed) was calculated.

### 3.7. Electron Microscopy

A 3-μL fibril sample was placed on a 300 mesh copper formvar/carbon grid (Electron Microscopy Science, Hatfield, PA, USA), and excess liquid was removed. The samples were negatively stained with 2% uranyl acetate, washed twice with H_2_O, and air-dried. Grids were analyzed on a Philips Tecnai T12 transmission electron microscope at 80 kV (FEI, Hillsboro, OR, USA).

## 4. Conclusions

We were able to demonstrate that AL proteins can present a wide range of thermal stability that is not associated with their germline donor sequence but rather with the location of their somatic mutations. The kinetics of amyloid formation are correlated with thermal stability at pH 7.4 for some of the proteins characterized in this study. Interestingly, AL-T03 is the most unstable protein ever characterized in our laboratory and, to the best of our knowledge, the most unstable AL protein ever reported in the literature. AL-T03 does not form amyloid fibrils at pH 7.4, and it forms a mixture of amorphous aggregates and amyloid fibrils at pH 2.0. AL-T03 comes from the only patient selected for this study that presented organ response, suggesting that there is a thermal stability threshold for amyloid formation and cytotoxicity in AL amyloidosis.
